# The prevalence and consequences of punitive time and attendance policies in the food and retail industries

**DOI:** 10.1080/13668803.2026.2689580

**Published:** 2026-06-18

**Authors:** Kess L. Ballentine, Meredith Slopen, Daniel Schneider, Kristen Harknett

**Affiliations:** a School of Social Work, Wayne State University, Detroit, MI, USA;; b School of Social Welfare, Stony Brook University, Stony Brook, NY, USA;; c Harvard University, Harvard Kennedy School, Cambridge, MA, USA;; d Sociology Department, University of California-Berkeley, Berkeley, CA, USA

**Keywords:** Job quality, service sector, attendance, workplace policy

## Abstract

Work time control and flexibility are beneficial for workers, but many low-income, hourly workers face rigid expectations to be available for all scheduled shifts without modification. This study examines an understudied dimension of work-time control: punitive time and attendance policies. Many companies apply these ‘point systems’ in which points are used as sanctions for time and attendance policy violations. When points accumulate, they lead to increasingly serious consequences with the potential to exacerbate inequality for workers with caregiving responsibilities or health problems. Recent prior research has relied on content analysis of points policies or on single-company surveys. In this paper, we provide the first large-scale examination of inequalities in point systems. We draw on novel survey data collected by the Shift Project from 4,209 service sector workers to document the prevalence and consequences of point systems in the U.S. We find that point systems affect more than 2 of 5 hourly workers at large service sector firms, and those in poor health are disproportionately penalized under point systems. Further, workers penalized through point systems have worse employment and well-being outcomes, raising concerns that point systems may compound disadvantages faced by workers with disabilities or chronic health conditions.

## Introduction

A large literature documents the precarity of modern employment for many workers, seen in the fissuring of traditional employment relationships and the rise of sub-contracting, outsourcing, franchising, and independent contracting (Weil, 2014). But, all is not well within traditional employment relations either. A growing literature in sociology, demography, economics, public health, and related fields has chronicled the rise of precarious working conditions, from stagnant wages (Cooper et al., 2019; Gravelle, 2020), to retrenchment in core benefits (Hacker, 2019), to unstable and unpredictable work schedules (Lambert, 2008; Schneider & Harknett, 2019). Especially in the case of the latter, these labor practices serve not only to minimize labor costs (Lambert, 2008) but also as levers of power and control for employers (Wood, 2020).

Some employers also exert control over workers through punitive time and attendance policies, sometimes called point systems. Point systems enumerate attendance-related policy violations, such as calling off or clocking in late, that translate to consequences, such as a certain number of points resulting in a written warning or termination (Bakst et al., 2020). Large companies argue that they need point systems to manage attendance, as they standardize, and often automate, consequences for time and attendance policy violations across large employers by being applied consistently across employees directly through payroll technology (Bakst et al., 2017; Brooks, 2024). However, these policies also have the potential to become systems of control and subjugation that negatively affect workers. The policies are enforced with varying levels of warning, severity, and consequences, but sometimes include punishment even for the use of leave time for legally protected activities, including when employees have earned such time through a collective bargaining agreement (Bakst et al., 2017, 2020; Ballentine, 2024). If these systems are used in low-wage industries such as the service sector, they could play a compounding role in the poverty traps in which many low-wage workers find themselves (Ballentine, 2024; Harknett et al., 2024).

Despite suggestions that a large portion of American workers may experience stress because their access to time off is being monitored by punitive time and attendance policies or point systems (Bakst et al., 2017, 2020; Matos & Galinsky, 2014; Miller et al., 2011), such policies, and particularly workers’ experiences of them, have rarely been empirically evaluated. Available prevalence estimates are more than a decade old, predate the introduction of new technologies related to scheduling, time keeping, and worker control, and offer little detail about the policies (Matos & Galinsky, 2014; Miller et al., 2011). We developed and fielded a new battery of survey items designed to capture front-line hourly service-sector workers’ experiences with punitive time and attendance policies at large firms in the United States. The retail and food service sector constitutes almost 20% of jobs in the U.S. economy (U.S. Bureau of Labor Statistics, 2025), and these are the quintessential bad jobs in the U.S. economy, characterized by low pay, precarious schedules, and few benefits (Kalleberg, 2011). Although point systems are not limited to the service sector (Bakst et al., 2020), these practices could have particularly serious implications for retail and food service workers, given their limited job autonomy and economic resources. We fielded our new survey module between September and November of 2024 to 4,209 hourly workers employed at 132 of the largest firms in the service sector in the U.S. We use these data to provide up-to-date evidence on the prevalence of punitive time and attendance policies as reported by hourly workers employed in the service sector. We also provide evidence on whether these policies are applied uniformly or whether they disproportionately affect certain groups of workers, such as caregivers or those with health conditions who may have greater need for schedule flexibility. We also present new evidence on how these point systems affect workers’ employment and health outcomes.

We find that punitive time and attendance policies are prevalent in the service sector, with nearly 43% of workers in our sample reporting a point system at work. Of these, 20% have accrued points and 19% report negative consequences, including worse schedules and blocked promotions. We find evidence of disparate impacts of these policies for workers in poor health, who are far more likely to have been sanctioned than those in good health, but do not find expected inequalities by parental status. Finally, we find that point sanctions are associated with increased intent-to-leave, job dissatisfaction, psychological distress, and unhappiness.

### Theoretical framework

Our study is informed by theory documenting the ‘ideal worker’ archetype and work/family border theory. When employers expect workers to follow the norms of the ideal worker, they expect workers to be fully committed to their work, to prioritize work over personal or family needs, and to reject any potential interruptions to their paid labor, regardless of any legitimate need for flexibility or accommodation (Abramovitz, 1996; Acker, 2006). Researchers have documented that violations of the ideal worker norm can expose workers to stigma, scrutiny, and sanctioning from their employer (Dodson, 2013; Scheiman & Glavin, 2008; Storer et al., 2020). Work/family border theory conceptualizes how employees and employers interact at the border between work and family (Clark, 2000; Kossek & Lautsch, 2012; Nipert-Eng, 1996, 2008). Work/family border theory suggests that ‘border keepers’, such as managers, determine the level of flexibility allowed at the border.

We posit that one way that employers enforce the ideal worker at the work/family border is by penalizing any violation of rigid border crossing norms (e.g. calling off vs coming in, running late vs. being punctual; Ashforth et al., 2000; Clark, 2000). As such, point systems institutionalize employer expectations that employees transition to their employee role at specific, employer-determined times and durations (Ashforth et al., 2000). Thus, firms create a non-human, automated ‘border keeper’ via the point system to enforce strict work/family borders that are consistent with norms of the ideal worker. Based on this framework, we expect that workers with family caregiving responsibilities or health issues, whose need for flexibility to care for themselves or others violates the ideal worker norm, will be more likely to be punished at the border via punitive time and attendance systems. We also expect that the more frequently the worker violates border norms, the greater their separation from the ideal worker archetype, which may result in increasing sanctions by the employer and, by extension, stress for the worker.

## Background

### Time and attendance policies

Time and attendance policies set requirements for when employees arrive at and leave work, as well as the consequences for failing to meet these policies. When these policies are linked with employee disciplinary systems, they are considered punitive time and attendance policies. Such policies can include absence control policies, no-fault attendance policies, progressive discipline systems, or, more informally, point systems. These policies typically set a margin of error around the start time that enables employees to clock in within a few minutes before or after their start time without consequence. Additionally, they attach a firm-determined number of points to a set of ‘offenses’ or ‘occurrences’ that include (1) arriving outside the margin of error for arrival time (e.g. 3 min before or after technical start time), (2) leaving before their assigned clock-out time, or (3) calling off of work without adequate lead time or approval. Points for offenses accrue over a set period of time (e.g. a year) and lead to consequences that become progressively more serious, such as a verbal warning, a written warning, and termination.

These policies are a subject of debate, with employers arguing for their need and benefit, and worker advocates raising concerns about their unintended consequences. Employers argue that time and attendance policies are a necessary and basic human resource management tool to track absences and lateness that, over time, may be costly for businesses (Bakst et al., 2017). Moreover, some employers argue that such standardized systems promote equity by ensuring a regular schedule of consequences for violations of HR policies (see, for example, Atwork, 2025). However, a key concern about point systems is that they may inhibit workers from taking time off from work for good cause or legally protected reasons, such as when they are sick. Among low-wage workers, the limited available data examining their ability to access time off suggests that punitive time and attendance policies may be a significant barrier to using paid time off (Bakst et al., 2020; Ballentine, 2024; Williams, 2006). This may have negative implications. For example, presenteeism – or working when sick – has been shown to increase the circulation of communicable disease (Pichler et al., 2021). Point systems may also interfere with workers’ ability to attend medical appointments and fulfill household and caretaking responsibilities, ultimately leading to negative effects on worker health and well-being. These systems may also have similar negative effects on employers, including increased turnover (Piszczek et al., 2021) and reduced productivity (Boltz et al., 2023) from workers who are dissatisfied with their working conditions.

The U.S. advocacy group, A Better Balance, is responsible for two reports examining punitive time and attendance policies that helped identify such policies as a potential target for policy change. Specifically, their 2017 report, *Pointing Out: How Walmart Unlawfully Punishes Workers for Medical Absences*, surveyed 1,000 Walmart workers to show that a point system was punishing employees for using time off for illness and caregiving, reasons for time off from work that are protected by a range of state and federal policies (Bakst et al., 2017). Their 2020 report analyzed written time and attendance policies from 66 large U.S. employers to find that such policies limited workers’ ability to use legally allowable time off both in their construction and implementation (Bakst et al., 2020).

Besides these two reports, a single peer-reviewed article has been published on the policy, drawing from in-depth qualitative interviews with 21 urban healthcare workers (Ballentine, 2024). Workers in this study consistently raised point systems as one of the most harmful workplace policies affecting their ability to balance working while parenting school-age children. Further, contrary to employer claims that these systems advance equity in enforcing employment standards, this study found evidence that caregivers and those contending with structural discrimination were more affected by point systems and more likely to bear the worst consequences, such as having to change jobs or being fired due to earning too many points. Taken together, this small body of literature suggests that punitive time and attendance policies may be widespread and may add to employment instability while disproportionately affecting workers coping with their own or their family members’ illnesses and disabilities.

### Inequality in time and attendance policies

Based on our theoretical framework and prior research, we expect that punitive time and attendance policies may be particularly challenging for pregnant workers, caregivers, and people with disabilities who may require more absences from work (Bakst et al., 2020). Specifically, point systems may exacerbate inequalities by disproportionately disadvantaging people with chronic illness, disabilities, or caretaking responsibilities in part because such workers often require greater schedule flexibility and support to manage these additional needs (Bosma et al., 2020; Ireson et al., 2018; Spann et al., 2020). Research suggests that workers with disabilities and/or chronic health conditions face barriers to employment and retention in part due to employers’ concerns that these workers are expensive (Andersen et al., 2023; Bosma et al., 2021; Carolan et al., 2020; Nagtegaal et al., 2023). Workers in fair or poor health and those with caretaking responsibilities may need greater flexibility and accommodation at the work/family border and, therefore, may be less likely to be able to comply with ideal worker norms. As such, we hypothesize:

Hypothesis 1a: Compared to those without caretaking responsibilities, workers with caretaking responsibilities are more likely to incur points and consequences when working in firms with point systems.
Hypothesis 1b: Compared to workers in good, very good, or excellent health, workers in fair or poor health are more likely than their counterparts to incur points and consequences when working in firms with point systems.

### Punitive time and attendance policies and employment outcomes

Prior research shows the accrual of points can lead to workplace consequences, including receiving fewer hours, being assigned to a worse schedule, or being fired (Bakst et al., 2017; Ballentine, 2024). Within the precarious labor market, being fired or otherwise forced to find a new job due to a punitive time and attendance policy can put individuals at risk for even more precarious work (Ballentine, 2024). In general, we expect that workers employed by a firm with a point system will experience worse employment outcomes as a result of the point system, evidenced by worse job satisfaction and greater intent to leave than workers in firms without point systems. Further, due to the cumulative nature of the point system, when a worker has points or experiences consequences, compared to being unaffected, we expect their outcomes will worsen as they earn more points, which we differentiate as only earning points and no consequences versus earning points and consequences. As the intensity of the point system effect increases, we expect that workers will respond as they do to other documented workplace consequences with increased intent-to-leave and lower job satisfaction (Grimmond et al., 2024). Thus, if workers feel they are unable to meet employer expectations to perform as an ideal worker who respects rigid and impermeable work/family borders that we posit are enforced via point systems, we expect:

Hypothesis 2: Working in a firm with a point system or experiencing more point-system related sanctions, including points or consequences, is negatively associated with job satisfaction and intention to leave.

### Punitive time and attendance policies and well-being outcomes

Prior research builds a strong case for workers’ need for paid leave, which has been associated with better individual, familial, and community health as well as improved employee productivity and retention, while lacking access to paid leave has negative consequences (Goodman & Schneider, 2021; Lamsal et al., 2021; Peipins et al., 2012; Pichler et al., 2021; Slopen, 2023; Vander Weerdt et al., 2023). But theory and prior literature suggest that point systems may function as a barrier to taking needed or earned leave. Our theoretical framework suggests that taking time off from work is a violation of the performance of ideal workers because it indicates they are prioritizing outside obligations rather than work (Abramovitz, 1996; Acker, 2006). Prior research further suggests that when workers experience barriers to accessing paid leave, including an unsupportive workplace climate or perceived consequences of taking time off, such as losing money, being judged by supervisors, or letting down their coworkers, they fail to use all of their earned paid leave, which is the primary way U.S. workers are able to take off for illness or medical care (Ireson et al., 2018; Kuykendall et al., 2021; McNamara et al., 2012). As such, workers with employers who implement point systems may feel pressured to go to work when they or a family member are sick, avoid taking time off for necessary appointments, or experience stress from worrying about being even a few minutes late for work. By extension, lack of flexibility or access to paid leave puts workers at risk for worse well-being outcomes. To test this, we examine indicators of well-being, specifically psychological distress and happiness, that have been previously studied when examining connections between worker well-being and paid leave access (Stoddard-Dare et al., 2018).

Hypothesis 3: Working in a firm with a point system or experiencing more point-system related sanctions, including points or consequences, is associated with worse worker well-being outcomes, including greater psychological distress and less happiness.

## Methods

### Data and sample

Our study uses survey data collected by the Shift Project in the Fall of 2024 from 4,209 hourly workers employed in the service sector. Twice annually since 2017, the Shift Project has used Meta’s advertising platform to recruit hourly workers from large U.S. firms in the food and retail services sector to participate in cross-sectional Qualtrics surveys (Schneider & Harknett, 2022). The Shift Project survey captures information about working conditions for a large national sample of retail and food service workers employed at large U.S. firms (e.g. retail, big-box, grocery, pharmacy, delivery and fulfillment, fast food, and casual dining sectors). Although the Shift Project is a non-probability sample of workers, these data have been shown to closely replicate findings from two gold-standard probability data sources in the United States: the Current Population Survey and the National Longitudinal Survey of Youth 1997 (Schneider & Harknett, 2022). All study procedures are in compliance with the University Institutional Review Board.

The Shift Project survey wave in Fall of 2024 included a special module on punitive time and attendance policies (see Appendix 1) along with a standard set of questions related to job quality, demographics, economic security, health outcomes, and parenting. The survey was self-administered online in Qualtrics. As is typical in self-administered surveys, not all who begin the survey will complete it. In this case, of the 6,700 workers who began the survey, 63% progressed far enough in the survey to complete the module on punitive time and attendance policies, yielding our sample of 4,209 hourly worker survey respondents (Table 1). The data are weighted on age, gender, and race/ethnicity to represent the population of service sector workers captured in data from the American Community Survey (U.S. Census Bureau, 2026). Respondents tended to have a high school degree (40%) or some college (33%). Almost 40% of our sample reported fair or poor health, and 40% reported having children in their household. Notably, 78% reported that it is very difficult or somewhat difficult to cover expenses.

### Measures

#### Level of exposure to punitive time and attendance systems

Drawing on the limited available prior research on point systems (Bakst et al., 2017, p. 2020; Ballentine, 2024), we developed a new survey module to identify workers who are exposed to punitive time and attendance policies and to learn more about their experiences with these systems. Lawyers at the workplace justice organization, A Better Balance – who have represented workers affected by point systems – provided feedback on a draft of the module based on their knowledge of the policies. Questions from the module can be found in Appendix 1.

Our key predictors gauge whether or not a respondent works in a firm with a point system and whether they have received points and/or point-related consequences. First, workers self-report whether or not there is a point system at their workplace, to their knowledge. Specifically, workers are asked about whether there is a policy, system, or bank where employees lose or earn points, occurrences, demerits, or disciplinary charges (points) for being late, leaving early, or taking time off. From this measure, we created a binary variable for whether there was a point system in their workplace (0 = work in a firm with no point system, 1 = work in a firm with a point system).

The module then asks if workers themselves lost or earned a point in the past 12 months and, if so, explores the reasons for receiving the point and the consequences that the worker may have experienced as a result of point accrual. From this information, we created a categorical variable with four categories: those who worked at a firm with no point system, those who worked at a firm with a point system but reported no points accrued, those who worked at a firm with a point system and received some points but no consequences, and those who worked at a firm with a point system and received both points and a resulting consequence. This measure enabled us to consider the intensity of the effect of point systems in order to comprehensively test our second and third hypotheses.

#### Outcome variables

We measure two employment outcomes: intent to look for a new job within 3 months and job satisfaction. We created a binary intent-to-leave variable from responses to the question ‘Taking everything into consideration, how likely is it you will make a genuine effort to find a new job within the next 3 months?’ where responses of ‘very likely and somewhat likely’ were coded as 1 and ‘not at all likely’ was coded as 0. A binary job satisfaction measure was created based on the question ‘All in all, how satisfied would you say you are with your job at [employer]?’ where ‘very satisfied’ and ‘somewhat satisfied’ were coded as 1, and ‘not too satisfied’ and ‘not at all satisfied’ were coded as 0.

We also measure two self-reported measures of workers’ well-being over the last month: psychological distress and happiness. Psychological distress is measured using a dichotomous version of the Kessler-6 psychological distress scale, which asks respondents to indicate on a 5-point scale how they felt over the last 30 days related to six variables: nervous, hopeless, restless or fidgety, so depressed that nothing could cheer you up, that everything was an effort, and worthless (Kessler, 2003). Responses are then summed, and those with scores greater than or equal to 13 are considered to be experiencing psychological distress and coded as 1 (otherwise 0). Happiness was measured with a standard happiness question used in the General Social Survey that asks ‘Taken all together, how would you say things are these days? Would you say you are … ‘ with ‘very happy’ or ‘pretty happy’ being coded as 1 and ‘not too happy’ being coded as 0 (Davern et al., 2023).

#### Control variables

Models control for individual and employment characteristics. Individual characteristics include age, gender, race/ethnicity, cohabitation status, educational attainment, household size, presence of children in the household, and difficulty meeting monthly expenses. Based on the prior literature, we expect some protective factors to mitigate the effects of the point system on individual outcomes. Given their relative power in the workplace (Clark, 2000), managers may be less affected by point systems (Ballentine, 2024). Additionally, labor unions protect workers by improving job quality and providing due process to grieve perceived employer wrongdoings, such as inappropriate application of points (Hagedorn et al., 2016; Rothstein, 2016). We control for these protective factors in our model, as well as wage rate (hourly wage in US$) and full-time status (working more than 35 hours per week).

To test our hypotheses, we also stratify our analysis by parental and health statuses. Parental status was a binary variable from the survey question: ‘Do you have any children? These might be your biological children, step children, adopted children, or foster children.’ Using the single item self-rated health question, which asks ‘in general how would you rate your health ‘, we divided the 5-point scale responses of self-reported health to create two groups; the good health group included those who reported their health as ‘excellent,’ ‘very good,’ or ‘good,’ and those in the fair or poor health group indicated ‘fair’ or ‘poor’ (Strawbridge & Wallhagen, 1999).

### Analysis

Our analysis proceeds in three parts. First, we describe the prevalence of workers’ exposure to time and attendance systems at large service sector employers.

Second, Hypotheses 1a and 1b, respectively, predict that parents and workers in fair or poor health are more likely to experience repercussions from time and attendance systems. We estimate an OLS regression to understand the strength of the association between parenting and health status with (1) working in a firm with a points system and (2) accruing at least one point, controlling for individual and workplace factors. Finally, we estimate the association between a four-category measure of level of exposure to point systems (none, point system without having incurred points, point system with having incurred points but no additional negative consequences, and point system with having incurred points and additional negative consequences) and a set of job-related (Hypothesis 2) and well-being (Hypothesis 3) outcomes.

## Results

### Prevalence and experience of time and attendance policies

We first describe the prevalence of time and attendance systems. The first column of Table 2 provides information on the exposure to point systems, accrual of points, and experience of consequences resulting from accumulated points. Overall, we found that 43% of workers reported that their employer used a punitive time and attendance policy. Among those whose workplace used a point system, about 3 in 5 workers had no points, 1 in 5 had points without experiencing consequences, and 1 in 5 reported experiencing consequences as a result of accruing points. On average, 38.4% of those working in firms with a point system earned at least one point, with nearly two thirds reporting that they earned points for personal health reasons (63%). Transportation delays (37%), family health reasons (30%), and medical appointments (20%) were other commonly cited reasons (Table 2).

**Figure 1.**
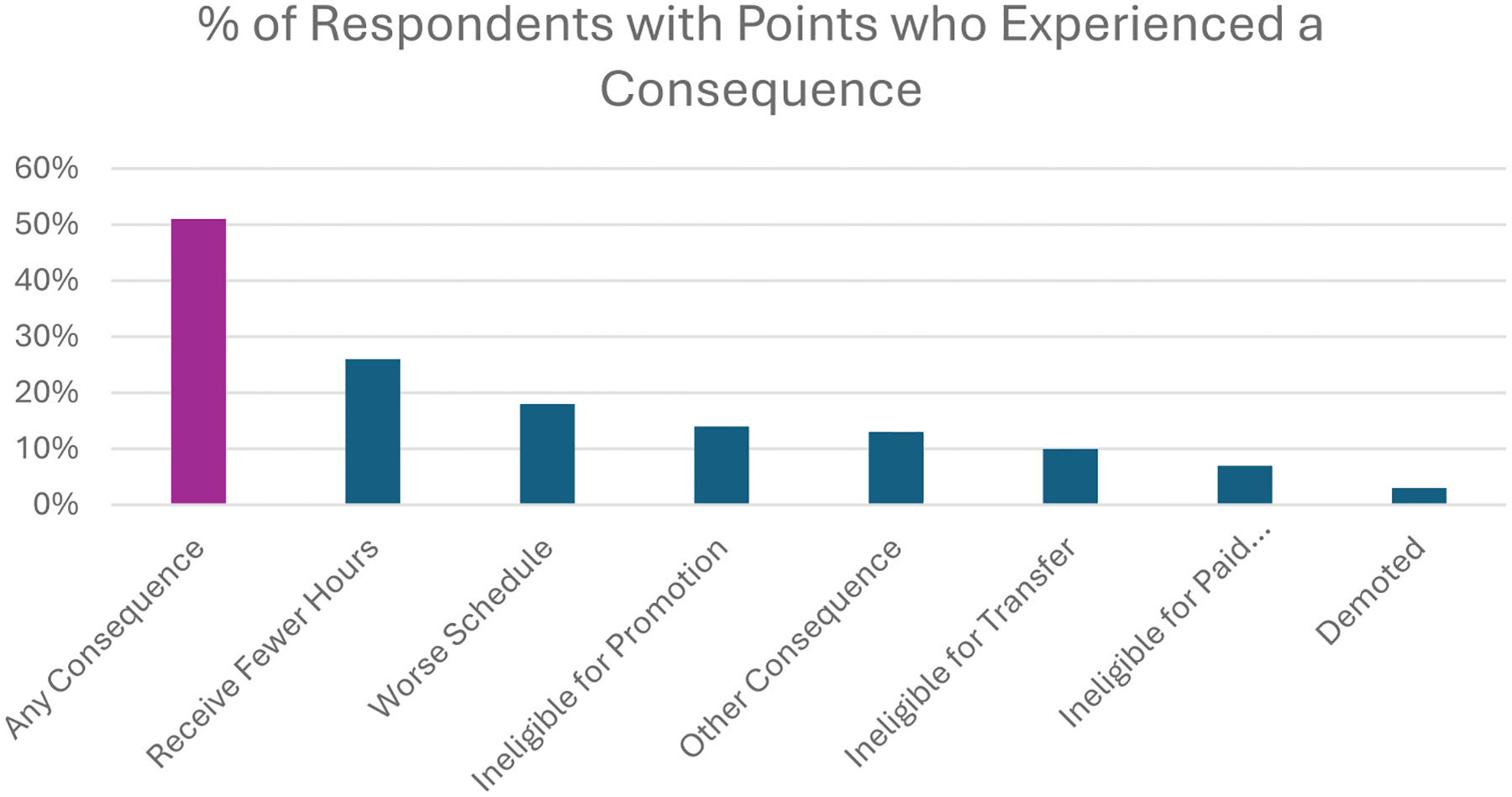
Consequences encountered with points. Notes: Shift Project, 2023. All proportions weighted to be representative by race, age, and gender using data from the American Community Survey.

**Figure 2.**
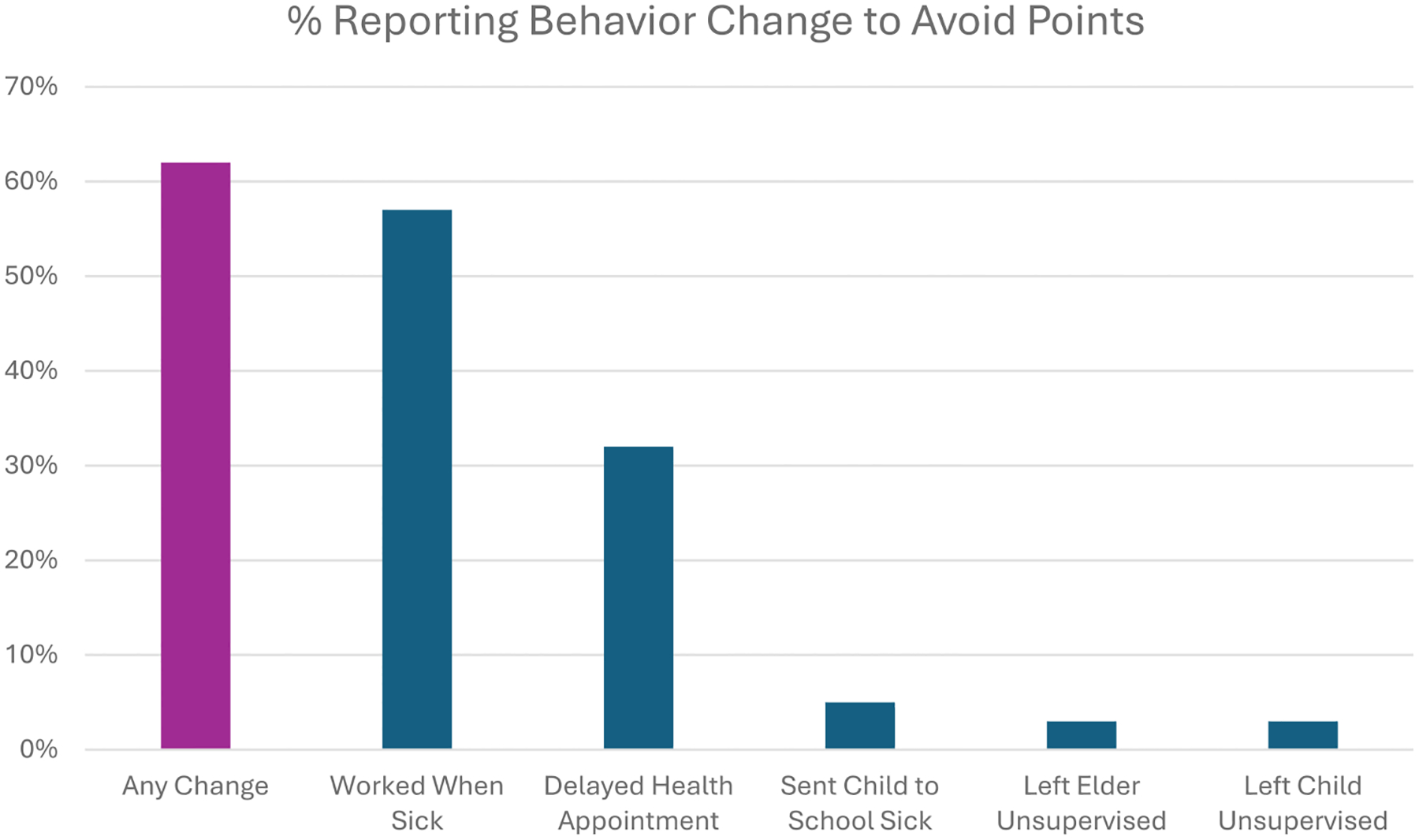
Changed behavior to avoid points. Notes: Shift Project, 2023. All proportions weighted to be representative by race, age, and gender using data from the American Community Survey.

While half of workers who had received a point reported no consequences beyond the point itself, the other half of workers who had received a point also experienced a consequence with a direct impact on their finances or family life. These consequences included that they had received fewer hours (25%), a less desirable schedule (17%), or that they became ineligible for promotion (14%), transfers (10%), or paid overtime (7%) as a result (Figure 1). Sixty-two percent of the workers who worked at a firm with a point system reported changing their behavior to avoid points (Figure 2). The primary choice workers made was to participate in presenteeism, working while sick, with 57% of workers at firms with point systems reporting working while sick in the previous year. One-third of workers at firms with point systems (32%) reported delaying a health appointment to avoid points. Other less common choices included sending a child to school sick (5%) or leaving an elder (3%) or a child (3%) unsupervised. Thus, though reported behaviors to avoid points might all have an impact on family health, the most common response to the risk of earning points was for the worker to sacrifice their own health.

### Heterogeneous exposures to time and attendance policies

Table 2 reports descriptively on heterogeneity in exposure and experiences of consequences from points systems by parental status and health status. On average, parents were less likely to work in positions subject to a point system, but those in fair or poor health were more likely to be subject to a point system at work. Comparing those with and without children, parents were slightly less likely to have any points, with or without consequences. However, when they had points, parents were statistically more likely than non-parents to report receiving points because of family health reasons, medical appointments, childcare emergencies, and school meetings. Conversely, those in fair or poor health were more likely to have points compared to those in good health and were twice as likely to have experienced a consequence as a result of those points. Those in fair or poor health were statistically more likely to accumulate points due to personal health reasons and medical appointments.

Table 3 shows results from linear regression models of associations between group status and experiences with point systems controlling individual and job characteristics (Appendix Table A1 shows the full results). Regression findings are consistent with descriptive findings. Specifically, we observe that respondents with children were significantly less likely to work in firms with a point system in place but were no more likely to have accrued points, compared with non-parents (Hypothesis 1a). Thus, we do not find evidence for Hypothesis 1a. In contrast, we do find evidence for Hypothesis 1b, as those reporting fair or poor health were both more likely to work in a firm with a point system than those in better health and significantly more likely to report having accrued points compared with those in good health (Hypothesis1b). These results suggest that point systems may lead to further disadvantages for workers facing health challenges.

### Consequences of time and attendance policies for worker outcomes

Consistent with our hypotheses, the second row of Table 4 shows that workers whose employers use a point system are more likely to be looking for a new job, less likely to be satisfied with work, more psychologically distressed, and less happy compared with their counterparts whose employers do not use a point system (see Appendix Table A2 for full results). Compared to those at workplaces without point systems, workers at firms with a point system are 7 percentage points more likely to be looking for a new job, 7 percentage points less satisfied with work, 4 percentage points more likely to be psychologically distressed, and 6 percentage points less likely to report being very or pretty happy.

In the lower panel of Table 4, we disaggregate the group that works at a firm with a point system into three more granular groups: (1) those who work at a firm with a point system but have not accrued points, (2) those who have received points but have not experienced consequences, and (3) those who have received points and experienced consequences. We compare each of these groups to their counterparts whose employers do not use a point system. This more granular comparison shows that the association between working for an employer who uses a point system and our outcomes is largely driven by workers who have received points and experienced consequences. Compared with workers at employers without a point system, workers who had experienced a point-related consequence were 26 percentage points more likely to intend to look for a new job, 30 percentage points less likely to be satisfied with their job, 21 percentage points more likely to report psychological distress, and 14 percentage points less likely to report being very or pretty happy. In contrast, those whose firm had a point system but had not accrued points were similar to those whose firms did not use a point system on each outcome. Those who had accrued points but had not experienced a consequence were also similar to those whose employers did not use a point system on intentions to look for a new job, job satisfaction, and psychological distress, but were significantly less likely to report being very or pretty happy. In sum, exposure to a point system is strongly associated with outcomes for those who have experienced a consequence for accrued points. Together these findings provide supportive evidence for Hypotheses 2 and 3.

## Discussion

Available evidence has suggested that punitive time and attendance policies serve as a barrier to workers accessing paid or unpaid time off that may have consequences for worker well-being and employment outcomes (Bakst et al., 2017, p. 2020; Ballentine, 2024). However, limited empirical data is available to understand the scope and impact of these policies, commonly referred to as point systems. To expand this nascent literature base and better understand workers’ experiences and barriers to leave, we draw on data from The Shift Project to present the first assessment of the use of point systems across a range of national retail and food service companies in the United States. Strikingly, we find that 43% of service-sector workers are subject to punitive attendance or point systems, suggesting that almost 11 million of the 25 million U.S. workers who are paid hourly and employed in the retail and food service sector are exposed to these policies (U.S. Bureau of Labor Statistics, 2025). We find that the primary reason workers accrue points under these systems is due to taking time off for health reasons, creating significant tension with public health goals. Given the scope of these policies and their public health implications, our study addresses a large gap in the literature on job conditions.

Our paper considered whether exposure to point systems is unequal and whether these policies have unequal consequences, in particular, for workers with caregiving responsibilities or those in poor health, each of whom may have a greater need for flexibility in their scheduled shifts. We found that parents are less likely to work in companies that use these policies, contrary to prior literature (Bakst et al., 2017; Ballentine, 2024). We suggest two potential explanations, though this finding merits further study. First, parents may actively avoid jobs with point systems because they anticipate needing flexibility. Alternatively, parents who are being managed out of jobs due to earning a high number of points may elect to leave to avoid consequences that may have implications for job-seeking, such as being fired (Ballentine, 2024). By extension, point systems may have implications for the number and types of jobs available to parents. Still, when parents were exposed to point systems and lost points, they were more likely to lose points for care-related reasons than non-parents, suggesting there may be differences in how point systems affect parents that merit further study.

More in line with previous findings, we found that those in fair or poor health are disproportionately more likely to earn points and experience consequences that limit their earning potential and may place them at risk of termination. This finding is consistent with our theoretical framework that predicted point systems may disproportionately affect workers who differ from the ideal worker archetype in that they cannot always maintain firm and inflexible work/family borders (Abramovitz, 1996; Acker, 2006; Ashforth et al., 2000) and has concerning implications for workers facing health challenges. Specifically, while paid leave can reduce the spread of communicable disease and increase the likelihood that working people use preventive care (Lamsal et al., 2021; Peipins et al., 2012), barriers to using leave, such as receiving points, may reduce the likelihood that people use their sick leave. Given that workers in fair or poor health may also have conditions that place them at higher risk of complications from communicable diseases, including COVID-19, the increased presenteeism associated with such systems might be seen as a particular risk for these populations and public health. The verity of this concern was emphasized by our finding that presenteeism and delaying medical appointments were the most common strategies workers used to avoid receiving points.

We hypothesized that greater exposure to point systems would have a negative impact on job and well-being outcomes. From an employment standpoint, point-related consequences were associated with greater turnover intention and lower job satisfaction, both of which may increase employer costs related to turnover and reduced productivity (Boltz et al., 2023; Piszczek et al., 2021). Indeed, we found that receiving a consequence through a point system was associated with higher levels of psychological distress and lower levels of happiness. Notably, we found that outcomes on these measures were only significant for those who had accrued points and attributed a workplace consequence to that point. The exception is happiness, where receiving a point – even without a consequence – lowered the likelihood that respondents were very or pretty happy by 13%. Thus, it may be possible that point systems have adverse effects on worker well-being even among workers who can comply with time and attendance policies.

The Shift Project’s novel survey data collection on punitive attendance policies provided a unique opportunity to study differences in the prevalence and consequences of these policies, but some limitations should be kept in mind. First, the Shift Project recruits survey respondents using advertisements on Facebook and Instagram targeted to workers who are employed at large retail and food service companies, which is a strategic and advantageous sample for studying time and attendance policies, but has limitations as a non-probability sample. Although the Shift Project data have been validated and found to replicate results from probability data sources (Schneider & Harknett, 2022), we caution that the estimates of the prevalence of exposure to point systems may not be representative of the service sector as a whole. Moreover, our sample is limited to the U.S., and we did not find related research from other countries, which may present an opportunity for future research. Second, in our analysis of differences in exposure to and consequences of point systems, our measure of caregiving is limited to parenting and fails to capture those who may be caring for older adults. Third, we limit our sample to those still employed, which may exclude workers who may have been most severely impacted by these point systems, specifically those who have been fired or quit. For this reason, we may underestimate the magnitude of associations between point systems and our job and well-being outcomes. Fourth, in contrast to prior literature (Bakst et al., 2017; Ballentine, 2024), we were surprised to find that parents had lower exposure to point systems than non-parents. Similarly, we found low rates of some caregiving behaviors (e.g. sending a child to school sick) being used to avoid points. It is possible that these behaviors may have been under-reported due to social desirability bias. Finally, although our findings are robust across models and to a large set of individual and job-level controls, our analyses of the job and well-being consequences arenot causal estimates. Further, the relationship between point systems and job and well-being outcomes could run in both directions; point systems may lead to outcomes like job dissatisfaction or psychological distress, but it is also possible that job dissatisfaction or psychological distress could increase the risk of accruing points. This risk is mitigated somewhat by the temporality of the survey questions, since we ask about point system exposure over the last 12 months, while outcomes are either at the time of survey completion for job outcomes or in the last 30 days for health outcomes.

With these limitations in mind, the results from this study have implications for researchers, labor rights advocates, and policymakers. These findings suggest that punitive time and attendance policies may exacerbate the risks of precarious labor for some workers, either increasing instability within their current employment or by pushing them out of a workplace and into a position with even lower job quality. Despite this, only a few publications have attempted to document their prevalence or impact. Additional research is necessary to understand the prevalence of these policies in other nations and industries, the full scope of their impact, and any causal mechanisms between points and worker outcomes. Still, these initial findings suggest that retaliation for taking time off, in this case via point systems, may be a critical area for work-family researchers to further explore to ensure workers can easily and consistently access legally allowable time off and flexibility.

Point systems may also merit more consideration by labor advocates and policymakers. As local and state governments work to expand access to paid sick leave and other types of protected leave, punitive time and attendance policies may undermine these policies by punishing workers even for protected time (Slopen et al., forthcoming; Bakst et al., 2020). Policymakers in New York state are the first to recognize the harmful role of point systems, having implemented the first legislation in the U.S. to limit the use of punitive time and attendance policies (State of New York, 2022). Given the wide usage of these policies across the U.S. and as other localities consider following New York’s lead, future research to understand the effects of these policies and of the New York policy change would be valuable.

Our study is the first large-scale study to examine time and attendance policies in the U.S. service sector. We demonstrate that these policies are common, disproportionately affect workers in poor health, and are associated with greater intention to leave, lower job satisfaction, worse psychological distress, and less happiness. Punitive time and attendance policies may exacerbate the risks of precarious labor for some workers, either increasing instability within their current employment or by pushing them out of a workplace and into a position with even lower job quality. This study expands a small but consistent literature base that suggests point systems may harm worker outcomes and merit additional attention by researchers and policymakers.

## Figures and Tables

**Table 1.** Sample descriptives (N = 4209).

Characteristic	%
Race/ethnicity	
White non-Hispanic	56
Black non-Hispanic	12
Hispanic	22
Other non-Hispanic	9
Relationship Status	
Married living with spouse	22
Living with partner	24
Not living with spouse or partner	51
Unknown cohabitation status	3
Gender	
Male	47
Female	53
Level of Education	
No degree or diploma	5
High school diploma/GED	40
Some college	33
Associate’s degree	10
Bachelor’s degree	11
Advanced Degree	2
Difficulty Covering Expenses	
Very difficult	30
Somewhat difficult	48
Not difficult	22
Manger	32
Union Member	11
Has Children	40
Fair or Poor Health	39
Job Outcomes	
Likely to look for a new job	50
Satisfied with work	77
Well-Being Outcomes	
Psychological Distress	35
Very or Pretty Happy	66
	*M*
Age	33.41

Notes: Shift Project, 2023. All proportions weighted to be representative by race, age, and gender using data from the American Community Survey. Some totals may not sum exactly to 100% due to rounding and weighting.

**Table 2.** Exposure to points system.

	Full population%	Has No children%	Has children%		Good health%	Fair or poor health%	
Workplace uses point system	42.8	45.4	39.0	*	40.8	47.4	**
	Among Those at Workplaces with Point Systems						
Does not have any points	61.7	59.6	65.3		67.7	53.6	**
Has points, no consequences	19.5	20.5	17.7		19.3	19.7	
Has points, experienced consequences	18.9	20.0	17.1		13.0	26.7	**
Reasons for losing points							
Personal health reasons	62.9	64.2	60.0		55.4	70.3	**
Transportation delay	37.3	42.8	26.3	**	32.9	40.5	†
Family health reason	30.3	27.0	36.6	*	29.5	29.9	
Medical appointment	19.6	16.8	24.4	*	15.7	22.2	*
Childcare emergency	9.9	2.1	24.6	**	7.5	11..9	†
Child’s school meeting	4.68	0.65	11.73	**	2.37	6.53	*
Other reason	23.0	23.7	21.5	**	23.8	21.4	
Observations	4209	2047	2110		2325	1559	

Notes: Shift Project, 2023. All proportions weighted to be representative by race, age, and gender using data from the American Community Survey. The differences in analytic sample size is due to differences in item non-response. Significant differences by subgroups indicated by †*p*<0.1, *p<0.05, **p<0.01.

**Table 3.** Association between exposure to points – hypothesized variables.

	Works in firm with point system	Any points if working in firm with point system
Has children	−0.09** (0.03)	−0.05 (0.04)
Fair or poor health	0.06* (0.02)	0.13* (0.03)
Observations	3370	1389

Notes: Shift Project, 2023. Linear regression models. Robust standard errors in parentheses.

The comparison group is respondents who work in a firm with no point system (column 1) and those who have not accrued points (column 2). All models control for individual and job characteristics and are weighted to be representative by race, age, and gender using data from the American Community Survey. The differences in analytic sample size are due to differences in item non-response for each dependent variable. Respondents who are missing on one or more covariates are not included in the analysis.

* *p*<0.05,

** *p*<0.01.

**Table 4.** Association between point systems and job and well-being outcomes.

	Looking for a new job	Satisfied with work	Psychological distress	Very or pretty happy
Firm does not use a point system	(ref.)	(ref.)	(ref.)	(ref.)
Firm uses point system	0.07** (0.02)	−0.07** (0.02)	0.04* (0.02)	−0.06** (0.02)
Observations	3514	3517	3346	3386
Works in a firm with no point system	(ref.)	(ref.)	(ref.)	(ref.)
Works in firm with point system, no points	0.01 (0.02)	−0.01 (0.02)	0.00 (0.02)	−0.01 (0.02)
Has points, no consequences	0.05 (0.04)	−0.02 (0.04)	−0.00 (0.04)	−0.13** (0.04)
Has points, experienced consequences	0.26** (0.03)	−0.30** (0.04)	0.21** (0.04)	−0.14** (0.04)
Constant	0.50** (0.08)	0.84** (0.08)	0.04 (0.08)	0.94** (0.09)
Observations	3474	3477	3307	3346

Notes: Shift Project, 2023. Linear regression models. All models control for individual and job characteristics and are weighted to be representative by race, age, and gender using data from the American Community Survey. Robust standard errors in parentheses. The differences in analytic sample size is due to differences in item non-response for each dependent variable. Respondents who are missing on one or more covariate are not included in the analysis.

* *p*<0.05,

** *p*<0.01.

## Appendix

### Appendix 1: module questions

1. How difficult is it to report being late, leaving early, or taking time off?

Likert- Very difficult to not difficult

Likert scale: # levels consistent with other questions in the survey.

2. At your job, is there a policy, system, or bank where employees lose or earn points, occurrences, demerits, or disciplinary charges for being late, leaving early, or taking time off?

Yes

No

DK/REF

Skip logic: Continue if Q2=yes or don’t know. If Q2=no, skip to the end of the module.

3. In the past 12 months, have you lost or earned a point, occurrence, demerit, or disciplinary charge at work for being late, leaving early, or taking time off for any reason?

Yes/no/dk

Skip logic: Continue if Q3=yes or don’t know; If Q3=no, skip to Q6

4. In the past 12 months, have you lost or earned a point, occurrence, demerit, or disciplinary charge at work for being late, leaving early for any of the following reasons?

(Mark all that apply)

1. I was sick or injured, including mental health needs
2. I had to care for an ill family member or loved one
3. I attended a medical or mental health appointment for myself or a loved one
4. I attended a meeting at my child’s school
5. I had a childcare emergency/unexpected closure
6. I had a transportation delay (e.g. bus running late or car trouble)
7. Another reason (specify)
8. None of these

5. In the past 12 months, have you experienced any of the following consequences due to having a certain number of time or attendance-based points, occurrences, demerits, or disciplinary charges on your record?

(Mark all that apply)

1. Got a worse schedule
2. Scheduled for fewer hours
3. Ineligible for paid overtime
4. Ineligible for a promotion
5. Got demoted
6. Ineligible for transfer within the company
7. Another consequence (specify)
8. None of these

6. If you earn a certain number of time or attendance-based points, occurrences, demerits, or disciplinary charges on your record, can that result in being fired?

Yes/no/DK

7. How much do managers/supervisors control whether these policies are enforced at your workplace?

A great deal

Somewhat

A little

Not at all

8. If you feel you were given or lost a point, occurrence, demerit, or disciplinary charge unfairly, how difficult is it to appeal it?

very difficult to not difficult

9. In the past 12 months, have you done any of the following to avoid losing or earning a point, occurrence, demerit, or disciplinary charge?

(Mark all that apply)

1. Left a child unsupervised at home
2. Left an elder unsupervised at home
3. Had to delay or reschedule healthcare for yourself or a loved one
4. Gone to work sick
5. Sent a child to school/daycare sick
6. None of these

**Appendix Table A1.** Association between exposure to points – full controls.

Characteristic	Works in firm with point system *Coefficient (SE)*	Any points if working in firm with point system *Coefficient (SE)*
Age		
18–24	Ref.	Ref.
25–54	−0.00 (0.03)	−0.06 (0.04)
54–64	0.03 (0.04)	−0.10† (0.06)
65+	0.05 (0.05)	−0.10 (0.07)
Female	−0.03 (0.02)	−0.04 (0.03)
Race		
White, non-Hispanic	Ref.	Ref.
Black, non-Hispanic	−0.01 (0.05)	−0.03 (0.07)
Hispanic	0.00 (0.03)	−0.04 (0.04)
Other, non-Hispanic	0.06 (0.04)	0.03 (0.06)
Relationship status		
Married	Ref.	Ref.
Cohabiting, not married	−0.00 (0.03)	0.02 (0.05)
Single	−0.02 (0.03)	−0.03 (0.04)
Highest level of education		
Less than high school	−0.06 (0.09)	0.06 (0.10)
High school degree	0.04 (0.08)	0.19** (0.08)
Some college	0.07 (0.08)	0.15* (0.08)
Associate’s degree	0.06 (0.08)	0.20** (0.09)
Bachelor’s degree	0.11 (0.08)	0.16† (0.09)
Advanced degree	Ref.	Ref.
Number in household	0.00** (0.00)	−0.00** (0.00)
Parent	−0.09** (0.03)	−0.05 (0.04)
Fair or poor health	0.06* (0.02)	0.13** (0.03)
Difficulty covering expenses		
Very difficult	0.16** (0.03)	0.14** (0.05)
Somewhat difficult	0.09** (0.03)	0.05 (0.04)
Not difficult	Ref.	Ref.
Full time	0.04 (0.02)	−0.06t (0.04)
Hourly wage		
$0-Minimum wage	−0.17** (0.06)	−0.30** (0.08)
Minimum wage – $15	−0.11** (0.02)	−0.16** (0.04)
Greater than $15	Ref.	Ref.
Union member	0.05 (0.04)	−0.06 (0.05)
Manger	−0.10** (0.03)	−0.21** (0.04)
Constant	0.37** (0.08)	0.39** (0.09)
Observations	3370	1389

Notes: Shift Project, 2023. Linear regression models. Robust standard errors in parentheses.

The comparison group is respondents who work in a firm with no point system (column 1) and those who have not accrued points (column 2). All models control for individual and job characteristics and are weighted to be representative by race, age, and gender using data from the American Community Survey. The differences in analytic sample size are due to differences in item non-response for each dependent variable. Respondents who are missing on one or more covariates are not included in the analysis.

† *p*<0.1,

* *p*<0.05,

** *p*<0.01.

**Appendix Table A2.** Association between point systems and job and well-being outcomes.

Characteristic	Looking for a new job *Coefficient (SE)*	Satisfied with work *Coefficient (SE)*	Psychological distress *Coefficient (SE)*	Very or pretty happy *Coefficient (SE)*
Point system exposure				
No point system in workplace	Ref.	Ref.	Ref.	Ref
Works in firm with point system, no points	0.01 (0.02)	−0.01 (0.02)	0.00 (0.02)	−0.01 (0.02)
Has points, no consequences	0.05 (0.04)	−0.02 (0.04)	−0.00 (0.04)	−0.13** (0.04)
Has points, experienced consequences	0.26** (0.03)	−0.30** (0.04)	0.21** (0.04)	−0.14** (0.04)
Age				
18–24	Ref.	Ref.	Ref.	Ref.
25–54	−0.12** (0.03)	0.02 (0.02)	−0.08** (0.03)	−0.00 (0.03)
54–64	−0.22** (0.04)	0.09 (0.03)	−0.19** (0.03)	0.06† (0.04)
65+	−0.33** (0.04)	0.07* (0.03)	−0.21** (0.04)	0.12** (0.04)
Female	−0.03† (0.02)	0.04† (0.02)	0.04† (0.02)	−0.02 (0.02)
Race				
White, non-Hispanic	Ref.	Ref.	Ref.	Ref.
Black, non-Hispanic	0.17** (0.04)	0.06 (0.04)	−0.01 (0.04)	−0.05 (0.04)
Hispanic	0.05 (0.03)	0.04† (0.02)	0.00 (0.03)	−0.04 (0.03)
Other, non-Hispanic	0.08* (0.04)	−0.03 (0.04)	0.03 (0.04)	−0.02 (0.04)
Relationship Status				
Married	Ref.	Ref.	Ref.	Ref.
Cohabiting, not Married	0.01 (0.03)	0.00 (0.02)	−0.01 (0.03)	−0.00 (0.03)
Single	0.03 (0.03)	−0.01 (0.02)	0.02 (0.03)	−0.09* (0.03)
Highest Level of Education				
Less than High School	−0.22** (0.09)	0.00 (0.08)	0.18* (0.08)	0.00 (0.09)
High School Degree	−0.18** (0.07)	0.01 (0.07)	0.12† (0.07)	0.01 (0.08)
Some College	−0.19** (0.07)	0.00 (0.07)	0.08 (0.07)	−0.01 (0.08)
Associate’s Degree	−0.15* (0.08)	0.05 (0.07)	0.07 (0.07)	0.06 (0.08)
Bachelor’s Degree	−0.09 (0.08)	−0.02 (0.07)	0.07 (0.08)	0.04 (0.08)
Advanced Degree	Ref.	Ref.	Ref.	Ref.
Number in Household	0.00** (0.00)	0.00 (0.00)	0.00** (0.00)	0.00** (0.00)
Parent	−0.05† (0.03)	0.04† (0.02)	−0.08** (0.02)	0.04 (0.02)
Difficulty Covering Expenses				
Very Difficult	0.29** (0.03)	−0.25** (0.03)	0.41** (0.03)	−0.39** (0.03)
Somewhat Difficult	0.13** (0.03)	−0.07 (0.02)	0.21** (0.02)	−0.12 (0.02)
Not Difficult	Ref.	Ref.	Ref.	Ref.
Full Time	0.00 (0.02)	−0.01 (0.02)	0.04 (0.02)	−0.03 (0.02)
Hourly Wage				
$0–Minimum Wage	0.02 (0.05)	−0.00 (0.05)	0.03 (0.05)	0.08 (0.05)
Minimum Wage – $15	0.04 (0.02)	0.05* (0.02)	0.03 (0.02)	−0.03 (0.02)
Greater than $15	Ref.	Ref.		
Union Member	−0.10** (0.03)	0.07* (0.03)	−0.07* (0.03)	0.02 (0.03)
Manger	0.01 (0.03)	−0.02 (0.02)	0.03 (0.02)	−0.00 (0.02)
Constant	0.56** (0.08)	0.83** (0.08)	0.10 (0.08)	0.92** (0.09)
Observations	3474	3477	3477	3346

Notes: Shift Project, 2023. Linear regression models. Robust standard errors in parentheses.

All models control for individual and job characteristics and are weighted to be representative by race, age, and gender using data from the American Community Survey. The differences in analytic sample size are due to differences in item non−response for each dependent variable. Respondents who are missing on one or more covariates are not included in the analysis.

† *p*<0.1,

* *p*<0.05,

** *p*<0.01.
